# Developing a Formulation Strategy Coupled with PBPK Modeling and Simulation for the Weakly Basic Drug Albendazole

**DOI:** 10.3390/pharmaceutics15041040

**Published:** 2023-03-23

**Authors:** Harsh Shah, Kushal Shah, Bhavin Gajera, Rutesh H. Dave, David R. Taft

**Affiliations:** 1 Invagen, A Cipla Subsidiary, Hauppauge, NY 11788, USA; 2 Takeda Pharmaceuticals International Inc., Cambridge, MA 02139, USA; 3 Impel NeuroPharma, Seattle, WA 98119, USA; 4 Division of Pharmaceutical Sciences, Arnold & Marie Schwartz College of Pharmacy and Health Sciences, Long Island University, Brooklyn, NY 11201, USA; 5 Samuel J. and Joan B. Williamson Institute for Pharmacometrics, Division of Pharmaceutical Sciences, Arnold & Marie Schwartz College of Pharmacy and Health Sciences, Long Island University, Brooklyn, NY 11201, USA

**Keywords:** PBPK modeling, absorption modeling, model transfer experiment, Simcyp^™^, parameter sensitivity, precipitation kinetics, supersaturation, formulation development

## Abstract

Albendazole (ABZ) is a weakly basic drug that undergoes extensive presystemic metabolism after oral administration and converts to its active form albendazole sulfoxide (ABZ_SO). The absorption of albendazole is limited by poor aqueous solubility, and dissolution is the rate-limiting step in the overall exposure of ABZ_SO. In this study, PBPK modeling was used to identify formulation-specific parameters that impact the oral bioavailability of ABZ_SO. In vitro experiments were carried out to determine pH solubility, precipitation kinetics, particle size distribution, and biorelevant solubility. A transfer experiment was conducted to determine the precipitation kinetics. A PBPK model for ABZ and ABZ_SO was developed using the Simcyp™ Simulator based on parameter estimates from in vitro experiments. Sensitivity analyses were performed to assess the impact of physiological parameters and formulation-related parameters on the systemic exposure of ABZ_SO. Model simulations predicted that increased gastric pH significantly reduced ABZ absorption and, subsequently, ABZ_SO systemic exposure. Reducing the particle size below 50 µm did not improve the bioavailability of ABZ. Modeling results illustrated that systemic exposure of ABZ_SO was enhanced by increasing solubility or supersaturation and decreasing the drug precipitation of ABZ at the intestinal pH level. These results were used to identify potential formulation strategies to enhance the oral bioavailability of ABZ_SO.

## 1. Introduction

Physiologically-based pharmacokinetic (PBPK) modeling has been widely adopted as an experimental approach for predicting the pharmacokinetic behavior of a drug in humans by using preclinical data. Applications of PBPK modeling include in vitro–in vivo correlation (IVIVC) [1,2,3,4], drug–drug interaction assessment [5,6,7], and in vitro–in vivo extrapolation (IVIVE) [8,9,10,11]. Additionally, PBPK modeling has been utilized for oral formulation development in order to mitigate or minimize the risks of bioequivalence (BE) failures. Biorelevant dissolution, using media that mimics the composition of gastrointestinal fluids, coupled with PBPK modeling has become a reliable tool in predicting and supporting pharmaceutical development, especially for poorly soluble drugs [12,13].

Bioavailability enhancement of low soluble drugs requires the development of supersaturated formulation strategies, such as nanoparticle amorphous solid dispersions (ASDs), lipid formulations, and cyclodextrin complexes [14,15,16,17]. These techniques can help facilitate the selection of a superior formulation based on in vitro experiments, yet questions remain regarding whether in vitro performance can directly translate into in vivo behavior. As a result, formulation scientists have turned to PBPK absorption modeling to predict the in vivo performances of the lead formulations under consideration. Over the past several years, researchers have used in vitro biorelevant dissolution testing, in conjunction with PBPK modeling, to predict the systemic exposure for the amorphous solid dispersion (ASD) formulations under evaluation [12,13] and to probe the potential food effects of ASD formulations [18].

Characterization of ASDs, with respect to supersaturation and precipitation kinetics, and incorporating these parameters into PBPK absorption models allow for more realistic and reliable predictions of the in vivo behaviors of these formulations. Precipitation kinetics is a critical determinant for absorption of a weak base low soluble drug, and researchers have relied on in vitro transfer model experiments to mimic the in vivo transit of drugs from the stomach into the intestine, where precipitation is inevitable, thereby, limiting the amount of the drug that is available for systemic absorption. This in vitro experimental approach has proved to be an important screening tool for oral formulations of weak base drugs and it has also been used to inform PBPK model development [9,18,19,20].

In addition to its demonstrated utility in predicting the in vivo performance for oral formulations of low soluble compounds, several researchers have used PBPK modeling to guide formulation development and optimization. For example, Kuentz demonstrated the utilization of drug absorption modeling as a rationale for the formulation selection of several investigational compounds by performing parameter sensitivity analysis to evaluate the impact of dose and particle size on pharmacokinetic parameters [21]. Likewise, Kesisoglou and Mitra, employing a Quality by Design approach coupled with absorption modeling to inform formulation development, used sensitivity analysis to evaluate the impact of various model parameters on the bioavailability of five novel compounds at different stages of their development [22]. These investigations support the application of PBPK absorption modeling to help guide formulation development for a low-soluble drug.

Albendazole (ABZ), a small molecule benzimidazole derivative, was first marketed in 1982 as an anthelmintic drug for clinical use. It is on the World Health Organization’s list of essential medicines [23]. After oral administration, ABZ undergoes extensive presystemic metabolism, converting into its active primary metabolite, albendazole sulfoxide (ABZ_SO), which is responsible for the drug’s activity. ABZ is a weak base and has been categorized as a BCS class II (low solubility and high permeability) compound. Low oral systemic exposure to ABZ_SO has been primarily attributed to the low solubility of ABZ and its precipitation in the small intestine [24].

The current research explored the capability of PBPK absorption modeling to guide the formulation development of albendazole. To the best of our knowledge, there is no research available where the capabilities of PBPK absorption modeling have been explored with albendazole. Based on its weak basic nature, in vitro transfer model experiments were utilized to determine the precipitation kinetic parameters upon the transfer of the drug from the stomach to the small intestine. These parameters, along with data generated from solubility studies, were used to inform PBPK modeling. This study aimed to investigate and understand the important physiologic and formulation-related parameters affecting ABZ oral absorption and the systemic exposure of its active metabolite, ABZ_SO, using the Simcyp™ Simulator. The results enabled the identification of specific potential formulation techniques, which can enhance ABZ_SO bioavailability and maximize efficacy by using an approach that can reduce the resources and time spent in various phases of drug development.

## 2. Materials and Methods

### 2.1. Materials

Albendazole (ABZ) USP (Lot No. ASA000124A), sodium hydrogen phosphate (Na_2_HPO_4_, Lot No. BJ2212), and sodium dihydrogen phosphate (NaH_2_PO_4,_ Lot No. FE0043) were purchased from Spectrum Chemical (New Brunswick, NJ, USA). A marketed immediate-release tablet formulation of albendazole (400 mg, Lot No. A6AGS035) was purchased from Mankind Pharmaceuticals (Maharashtra, India). Biorelevant media powder (Fasted State Simulated Gastric Fluid (FaSSGF)/Fasted State Simulated Intestinal Fluid (FaSSIF)/Fed State Simulated Intestinal Fluid (FeSSIF), Lot. No. FFF-0519-B) was purchased from Biorelevant Company (London, UK). Methanol and concentrated hydrochloric acid (36.5–38.0% *w*/*w*) were purchased from Sigma-Aldrich (St. Louis, MO, USA). All chemicals and reagents used in this research were of analytical grade.

### 2.2. Solubility Measurement

The equilibrium solubility of ABZ was measured by the shake flask method at 37 ± 2 °C in different pH solution systems: 0.1 N HCl (pH 1.20 ± 0.01 and pH 2.0 ± 0.01), 0.02 M acetate buffer (pH 4.5 ± 0.02), and 0.05 M phosphate buffer (pH 6.8 ± 0.01). Solubility was also determined in FaSSGF (pH 1.6 ± 0.01), FaSSIF (pH 6.5 ± 0.01), and FeSSIF (pH 5.0 ± 0.01) containing bile concentrations of 0.08 mM, 3 mM, and 15 mM, respectively [25]. For each experiment, an excess amount of ABZ (~10 mg) was dispersed in 3 mL of solution. A sample was collected after 72 h and filtered using 0.45 µm polytetrafluoroethylene membrane disc filters (Pall Corp., New York, NY, USA). The samples were analyzed using a validated HPLC method. All experiments were performed in triplicate. The pH solubility profile and biorelevant solubility data for ABZ were analyzed using Simcyp™ In Vitro Data Analysis (SIVA, Certara UK Ltd., Sheffield, UK) software in order to estimate intrinsic solubility and the micelle-buffer partition coefficient (log Km:w). These parameter values were incorporated into the PBPK model for ABZ.

### 2.3. Particle Size Distribution Analysis

Particle size distribution (PSD) data for the marketed formulation are not publicly available. Hence, as a reference, pure ABZ was used for these experiments. The particle size distribution of ABZ was measured by laser diffraction using a Mastersizer 3000 equipped with Aero S (dry dispersion feeder unit). The instrument software was used to calculate d_10_, d_50_, and d_90_, representing the volume percentage or portion of particles below the 10th, 50th, and 90th percentiles, respectively. PSD was characterized as a Weibull distribution function, and the data were analyzed to estimate the Weibull parameter values and mean radiuses, which served as the input parameters in the model development [26].

### 2.4. Transfer System Experiment to Determine Precipitation Kinetic Parameters

**Figure 1:**
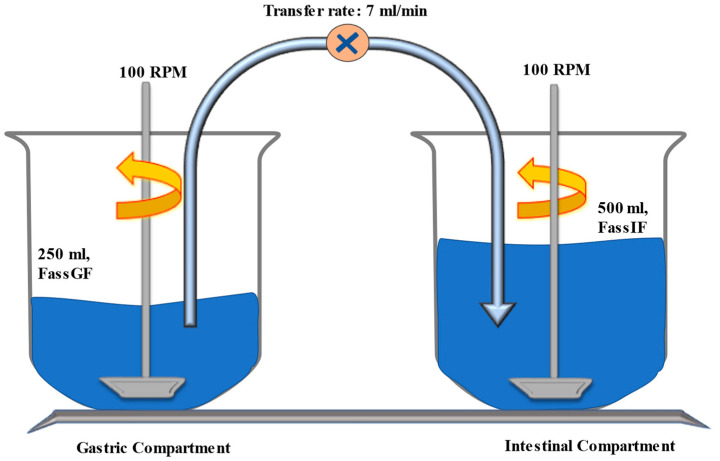
Transfer model setup using conventional dissolution apparatus, where FaSSGF represents the gastric compartment (donor compartment) and FaSSIF represents the intestinal compartment (acceptor compartment).

These experiments were carried out in order to characterize the precipitation kinetics of ABZ in a system designed to mimic the physiologic conditions in the GI tract as the drug moves from the stomach to the small intestine, where precipitation is inevitable and limits the availability of the soluble drug for absorption. To simulate the impact on solubility when the drug transits from the stomach into the intestine, the experimental system (Figure 1) consisted of two physiologically relevant compartments with allowances for modifications in transfer rate (representing the GI emptying rate), paddle speeds (representing the hydrodynamics inside the GI tract), and composition (representing fasted vs. fed conditions) [9,27,28,29,30]. A conventional dissolution system (Distek 5100, Distek Inc., North Brunswick Township, NJ, USA) with a USP-II paddle method was utilized. The gastric compartment contained FaSSGF media (250 mL, pH 1.6), and FaSSIF media (500 mL, pH 6.5), which constituted the intestinal compartment. FaSSIF was prepared with a higher buffer capacity to minimize pH changes after the complete transfer of the simulated gastric media. The paddle speed was maintained at 100 rpm for both compartments. The transfer was initiated after 30 min at a constant rate of 7 mL/min using an Econo™ Gradient pump (Bio-Rad Laboratories, Inc., Hercules, CA, USA). The following experiments were carried out: (a) 40 mg of free ABZ base (neat API) to maintain the sink condition in the gastric media. (b) Marketed immediate-release formulation (equivalent to 40 mg of ABZ) to maintain the sink condition and to determine the effect of excipients on promoting the supersaturation state. (c) Marketed immediate-release tablet formulation (400 mg of ABZ).

The gastric compartment was sampled at 5, 15, and 30 min, at which time the transfer to the intestinal compartment was initiated. Then, samples were collected from the intestinal compartment for an additional 90 min (32, 35, 40, 45, 50, 60, 75, 90, 105, and 120 min). Samples were filtered through 0.45 µm PTFE filters and immediately diluted with the mobile phase before quantitative analysis by HPLC. All dissolution experiments were performed in triplicate.

### 2.5. High-Performance Liquid Chromatography (HPLC) Analysis

Quantitative analysis of ABZ was performed by reverse-phase gradient high-performance liquid chromatography, in accordance with the method described in the United States Pharmacopoeia (USP) monograph for ABZ, with slight modification [31]. Analysis was conducted using Agilent ChemStation ^®^ analytical software on an Agilent 1100 system (Agilent Technologies, Palo Alto, CA, USA), equipped with a Zorbax SB-CN (4.0 mm × 25 cm, 5 µm) column (Agilent Technologies, Palo Alto, CA, USA). The temperature of the column was set at 25 °C and the samples were analyzed at 308 nm. The mobile phase consisted of acidified methanol: phosphate buffer (60:40% *v*/*v*). Acidified methanol comprised methanol and HCl (99:1), while the phosphate buffer consisted of 1.375% *w/v* monobasic sodium phosphate dissolved in deionized water. The injection volume was 20 µL, and the mobile phase flow rate was 1.5 mL/min. The detection wavelength was 308 nm. The method was validated according to USP guidelines. The assay was linear over a concentration range from 1 to 200 μg/mL The limits of detection and quantification were 0.24 μg/mL and 0.81 μg/mL.

### 2.6. PBPK Modeling

#### 2.6.1. Model Development

**Figure 2:**
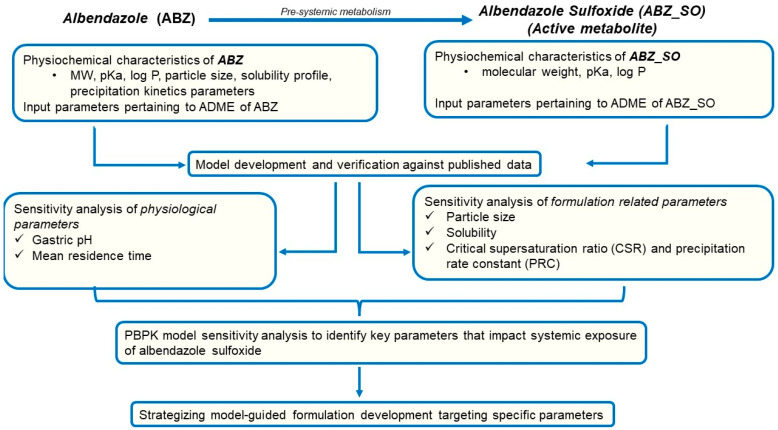
Flow chart for building a physiologically-based pharmacokinetic model for ABZ and ABZ_SO and strategizing formulation development.

PBPK modeling and simulation were performed using the Simcyp™ Simulator (Version 18, Certara, Sheffield, UK). The models for ABZ and its active metabolite ABZ_SO were built using the “middle-out” approach [32]. A summary of the PBPK model parameters for ABZ and ABZ_SO is provided in Table 1. ABZ distribution was modeled using the full PBPK model within Simcyp™ without optimization of the Kp scalar. Figure 2 represents a flow chart for building the PBPK model for ABZ and ABZ_SO and its application to strategize formulation development.

ABZ absorption was described using the Advanced Dissolution, Absorption, and Metabolism (ADAM) model. A literature-derived value for CaCO_2_ cell permeability was used to predict the in vivo absorption parameters fraction absorbed (fa) and the first-order absorption rate constant (ka) [39]. The measured particle size distribution was modeled as the Weibull distribution function in the ADAM diffusion layer model. Data from dissolution experiments were used to estimate intrinsic solubility and bile-salt micelle-mediated solubilization using SIVA. Micelle buffer partition coefficients (log Km:w) for ABZ, both in neutral and ionic species of ABZ, were estimated using observed solubilities in biorelevant media. Values for precipitation kinetics parameters were obtained from transfer model experiments. To model the clearance of ABZ, the “enzyme kinetics” option in Simcyp^TM^ was used and the parameter estimates for Vmax and Km CYP3A4- and flavin-containing monooxygenase 3 (FMO3)-mediated enzymatic pathways were obtained from the literature [36]. The FMO3 enzyme abundance in healthy volunteers was also obtained from the literature [40]. There is some portion of intestinal metabolism of ABZ because both enzymes are expressed in the intestine. The Simcpy^TM^ Simulator captures the intestinal metabolism based on the default abundance of both intestinal enzymes.

For the active metabolite, ABZ_SO, a minimal PBPK model was utilized. ABZ_SO absorption was modeled using ADAM. Permeability of the converted ABZ_SO was predicted using the mechanistic passive regional permeability predictor in Simcyp™, which calculates the drug permeability based on the log P of the drug. The distribution of ABZ_SO was modeled in Simcyp™ using the minimal PBPK model. Volume at steady state (Vss) and oral clearance were optimized using the Simcyp™ Parameter Estimation tool and data from a published clinical study.

#### 2.6.2. Model Verification in Healthy Human Volunteer Population

Clinically observed plasma profiles of ABZ and ABZ_SO were digitized from a published study using WebPlotDigitizer (Version 4.2, Austin, TX, USA), and the systemic exposure parameters (Cmax, AUC) were used to verify the model [41,42]. PBPK model simulations were performed using the Healthy Human Volunteer Population according to the design of the referenced clinical study: 10 subjects (6 male, 4 female), aged 21 to 44 years, single oral dose of albendazole (400 mg). Model verification was based on visual inspection of the simulated ABZ and ABZ_SO mean plasma versus time profiles compared to observed data, and the fold error (ratio of model predicted and observed values) for Cmax and AUC of ABZ and ABZ_SO.

#### 2.6.3. Sensitivity Analysis

The sensitivity analysis tool in Simcyp™ was used to assess the impact of physiologic (gastric pH and gastric MRT) and drug-specific (particle size, solubility, critical supersaturation ratio, and precipitation rate constant) model parameters on ABZ systemic exposure. PBPK model sensitivity for each parameter was carried out over a wide range of values (10 steps within each range) and assessed the impact on Cmax and AUC of ABZ_SO. The results were plotted as response surface 3D graphs using Originpro (Originlab, Northampton, MA, USA). Among the parameters tested were gastric pH (pH range: 1.2 to 4.5), mean gastric residence time (MRT, range: 0.2 to 2 h), particle size (range: 0.2 µm to 200 µm), and intrinsic solubility (0.012 mg/mL and 0.12 mg/mL). For the extreme solubility values (i.e., one-fold and ten-fold) the effect of varying the supersaturation and precipitation rates on ABZ-SO systemic exposure was also studied. This sensitivity analysis included a critical supersaturation ratio (2, 10, and 25) and a precipitation rate constant (0.004, 0.4, and 40 h-1). Sensitivity analysis of particle size was performed by fixing the initial value to the mean of the Weibull distribution (7.52 µm) and upper and lower bound values of distribution were assigned as the range for sensitivity analysis.

## 3. Results

### 3.1. pH Solubility Profile of Albendazole

Table 2 presents the ABZ solubility at different pH values in various media. This basic compound (pKa 2.8 and 10.3) has the highest ionization and maximum solubility (~500 µg/mL) at pH 1.2, with solubility decreasing drastically at higher pH values (e.g., ~5 µg/mL at pH 6.8). This decrease in solubility with an increase in pH can be attributed to the weak basic nature of ABZ, which has a pKa of 2.80. Aqueous solubility predictions using SIVA were in good agreement with the experimental values (R^2^ = 0.9771). The intrinsic solubility was estimated to be 0.012 mg/mL, and this parameter value was used in the PBPK model (Table 1). For biorelevant media experiments, solubility in FaSSGF was ~146 µg/mL, while solubility was 2-fold higher in FeSSIF (~20.5 µg/mL) compared to FaSSIF (8.2 µg/mL). These values were also well predicted by SIVA (R^2^ = 0.7448). Based on this biorelevant solubility data, SIVA estimated values for log K m:w (neutral) and log K m:w (ion) as 2.92 and 5.42, respectively. These bile-micelle partition coefficients for neutral and ionized species are required for the estimation of ABZ total solubility [43]

### 3.2. Particle Size Distribution

The measured d10, d50, and d90 for ABZ were 0.95 ± 0.10 µm, 4.1 ± 0.64 µm, and 22 ± 1.3 µm, respectively. These values were utilized to define the alpha and beta parameters of the Weibull distribution function, which were, then, used as an input for the ADAM model in Simcyp™.

### 3.3. Precipitation Kinetics

**Figure 3:**
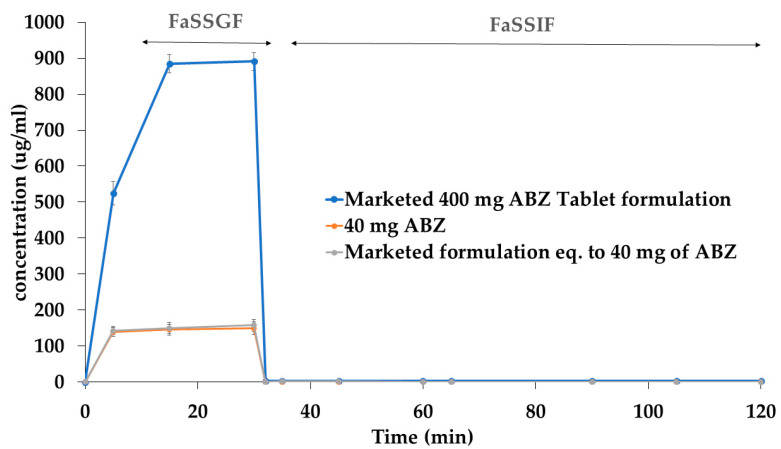
Transfer experiment results for ABZ (40 mg) and a marketed immediate-release tablet (40 mg and 400 mg).

Figure 3 depicts the concentration of ABZ over time during the transfer experiments. Data for pure ABZ (40 mg) and a marketed immediate-release formulation equivalent to 40 mg and 400 mg are presented in Figure 3. ABZ was completely dissolved within 30 min in FASSGF for both pure ABZ and the tablet formulation (40 mg dose) formulation. Upon transfer to FASSIF, the dissolved drug started to precipitate in the higher pH, and immediate precipitation was observed for both pure ABZ and the ABZ tablet (40 mg). For the marketed tablet (400 mg), approximately 50% (~900 µg/mL) of the drug dissolved within 30 min in FaSSGF, indicating saturation in the gastric media, followed by immediate precipitation upon transfer into the intestinal compartment. This immediate-release formulation failed to promote a supersaturation state and, therefore, had a minimal effect on delaying the precipitation of ABZ.

The parameter for the critical supersaturation ratio (CSR), an indicator of the degree of supersaturation, is calculated as follows:

(1) $$\mathrm{CSR}=\frac{\;\mathrm{Critical}\;\mathrm{supersaturation}\;\mathrm{concentration}}{\mathrm{Equilibrium}\;\mathrm{Solubility}}\;$$

The concentration of ABZ never exceeded the equilibrium solubility upon transfer to the intestinal compartment (FaSSIF), indicating immediate precipitation. Therefore, PBPK model parameter values for CSR and precipitation rate constant (PRC) were set to 1 and 1000 (1/h), respectively.

### 3.4. PBPK Model Verification

**Figure 4:**
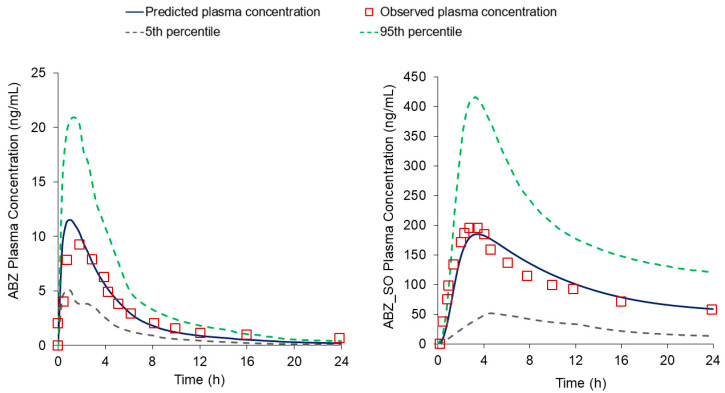
Plot of observed vs. predicted plasma concentration profile of ABZ (**left**) and ABZ_SO (**right**) in healthy volunteers.

The predictive performance of the PBPK model was demonstrated by comparing the simulated systemic exposure of ABZ and ABZ_SO to published clinical data [41]. As demonstrated in Figure 4, the model captured the mean simulated plasma profiles for both compounds. Table 3 compares the predicted and observed systemic exposure parameters (AUC and Cmax) for ABZ and its metabolite, and the fold errors ranged between 0.97 and 1.16. These results indicate that the PBPK model successfully predicted and recovered the pharmacokinetics of ABZ in healthy volunteers.

### 3.5. Sensitivity Analysis

A series of sensitivity analyses were carried out to evaluate the impact of various model parameters on the systemic exposure metrics (Cmax, AUC) of the active metabolite, ABZ_SO. This approach can potentially help guide the development of an improved oral formulation for the compound by identifying the critical parameters impacting in vivo performance. The results of these analyses are described below.

#### 3.5.1. Model Sensitivity to Gastric PH

**Figure 5:**
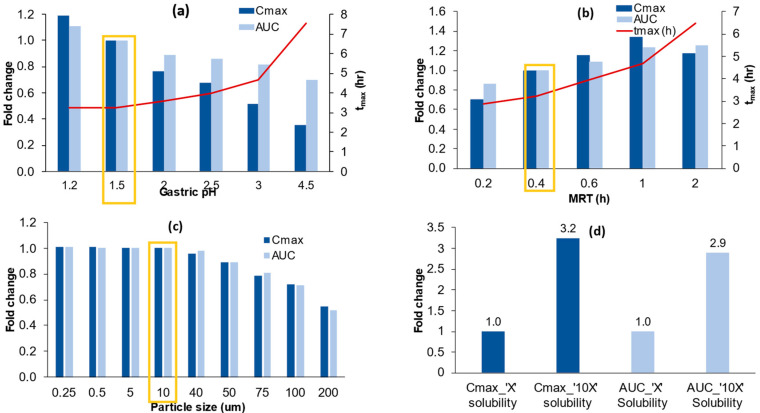
Simulated effect on pharmacokinetic parameters of changes in (**a**) gastric pH, (**b**) mean gastric residence time (MRT), (**c**) particle size, and (**d**) solubility (‘X’ and ‘10X’). The rectangular box represents the reference parameter used in the validation of the model.

Figure 5a illustrates the impact of gastric pH on ABZ_SO systemic exposure. Increasing the gastric pH above the reference pH value (1.5) was associated with reduced Cmax and AUC, with decreases of ~65% and ~30%, respectively, at pH 4.5. These changes were associated with an increase in the Tmax from 3.2 h (pH 1.5) to 7.5 h (pH 4.5). These sensitivity analysis results indicate that gastric pH is a critical attribute that cannot be overlooked during formulation development as it can lead to subtherapeutic exposure of ABZ_SO.

#### 3.5.2. Model Sensitivity to Gastric Mean Residence Time (MRT)

The sensitivity analysis showed that increasing gastric MRT was associated with higher systemic exposure, as demonstrated in Figure 5b. Compared to the reference value (0.4 h), increasing MRT to 1 h was associated with ~30% higher Cmax and ~24% higher AUC.

#### 3.5.3. Model Sensitivity to Particle Size

Figure 5c depicts the model-predicted impact of particle size on the rate and extent of absorption of ABZ_SO. Reducing the particle size below 40 µm (i.e., 0.25–40 µm) did not improve systemic exposure. At a particle size of 50 µm and higher, reductions in Cmax and AUC, as high as ~30% and 50%, were predicted, with minimal impact on Tmax, indicating a reduction in the ABZ_SO bioavailability.

#### 3.5.4. Model Sensitivity to Solubility and the Interplay between Degree of Supersaturation and Precipitation Rate

Figure 5d compares the PBPK model-predicted Cmax and AUC of ABZ_SO based on the experimentally determined solubility profile of ABZ and a hypothetical solubility that is ten-fold (10X) higher. Using the ‘10X’ input solubility profile, the model predicted a 200% enhancement in systemic exposure of the active metabolite, suggesting an increase in the absorption of ABZ by improving its equilibrium solubility.

**Figure 6:**
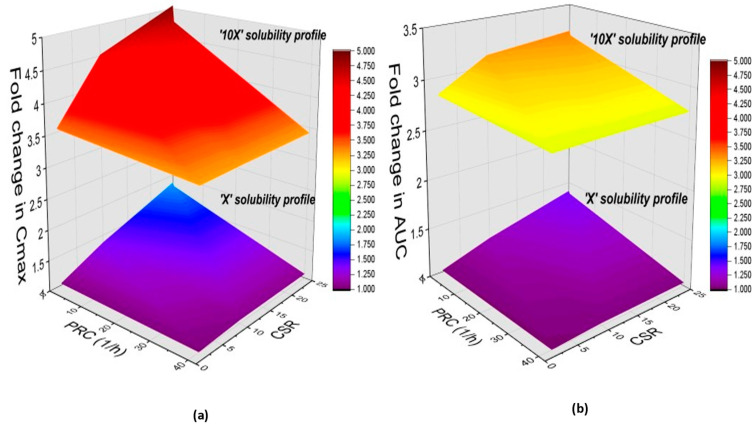
Simulated influence represented by the fold change of degree of supersaturation and precipitation rate on pharmacokinetic parameters: (**a**) Cmax and (**b**) AUC. Each figure represents the data of the ‘X’ and ‘10X’ solubility profiles.

Additionally, the impact of varying degrees of supersaturation over a range of precipitation rate constants on ABZ_SO Cmax and AUC is presented as response surface 3D graphs (Figure 6). This sensitivity analysis illustrates that a higher degree of supersaturation and slower precipitation rate of ABZ at the intestinal pH are critical parameters for improving systemic exposure of ABZ_SO. Model simulations with a CSR of 25 (highest value) and PRC of 0.004 h^−1^ (lowest value) were associated with increases in both AUC (80%) and Cmax (40%)—based on the experimentally determined solubility profile. When the analysis was repeated with the ‘10X’ solubility profile, Cmax and AUC increased by 400% and 200%, respectively.

## 4. Discussion

The general goal of oral drug administration is to deliver a sufficient amount of drug to the systemic circulation, where the drug can then distribute to its site of action. For a drug to reach systemic circulation, it must first absorb across the intestinal lumen, and this process depends on numerous factors, including aqueous solubility and membrane permeability. Once in the intestinal cell, the drug is susceptible to intestinal metabolism mediated by CYP3A4 and other enzymes before entering the bloodstream. Finally, the fraction of the dose absorbed into the bloodstream must first pass through the liver and escape hepatic metabolism (termed the “first-pass effect”) before becoming systemically available. The dependence of bioavailability (*F*) on these processes can be described by the following equation:

(2) $$F=f_{a}\times f_{g}\times f_{h}$$

where *f_a_* represents the fraction of the administered dose absorbed into the intestinal cell, *f_g_* is the fraction of the dose reaching the intestinal cell (*f_a_*) that escapes gut metabolism, and *f_h_* is the fraction of the dose absorbed into the bloodstream (*f_a_* × *f_g_*) that escapes first-pass hepatic metabolism.

ABZ is an anthelmintic drug used to treat systemic infections. The low absolute oral bioavailability (<5% in humans) of albendazole is due in part to extensive presystemic metabolism by the liver and small intestine [43], where ABZ is converted into its active metabolite ABZ_SO. However, systemic exposure to ABZ_SO largely depends upon the fraction of ABZ absorbed into the intestine (*f_a_*). Since ABZ is highly permeable, aqueous solubility is the critical determinant of ABZ absorption. Although increasing ABZ absorption would be expected to have a limited impact on ABZ bioavailability (due to extensive presystemic metabolism), it would be expected to increase the exposure to the active metabolite, ABZ_SO.

Solubility and dissolution testing using biorelevant media has become a critical tool for predicting the in vivo performance of oral dosage forms. This approach more reliably mimics gastric and intestinal fluid composition (ionic strength, viscosity, surface tension, and osmolality) [44]. PEARL (Pharmaceutical Education and Research With Regulatory Links, www.pearrl.eu), and OrBiTo (Oral Biopharmaceutics Tool project, an innovative medicines initiative) are examples of the efforts undertaken by academia and the pharmaceutical industry to develop new biopharmaceutics tools, which better predict in vivo performance, and thereby, facilitate drug development [45,46]. These approaches require a better understanding of processes that govern the absorption of orally administered drugs from the GI tract into the body. PBPK modeling, along with data from in vitro experiments, can help reduce drug development costs and provide a better understanding of the expected in vivo performance of the drug [47].

Research efforts aimed at creating drug formulations that can maintain higher solubility throughout the GI tract are increasing, mainly because there are a number of novel techniques available to achieve that goal. However, for a weak base drug such as ABZ, a formulation that allows for enhanced dissolution in the stomach can easily be counteracted by drug precipitation in the upper small intestine. When a weak base drug transits from the stomach into the duodenum, it is exposed to a ‘supersaturation state’. This can either lead to instantaneous drug precipitation or slow precipitation (at a first-order rate) after reaching the critical supersaturation concentration (CSC). Physiological factors (e.g., transfer rate, bile-salt concentration, and hydrodynamics at the GI lumen), physiochemical properties (e.g., pKa and crystallization tendencies), and formulation excipients can all influence the precipitation kinetics of a drug [16,28,31,48]. In this research, sensitivity analyses using PBPK modeling provided critical insight into the physiological and formulation-related variables that influence ABZ absorption after oral administration.

Several studies reported decreased and varying absorption of weakly basic drugs under exposure to high gastric pH conditions leading to subtherapeutic exposure. This phenomenon has been attributed to the slow and incomplete dissolution of these drugs when stomach pH is high [49,50,51]. Elevated gastric pH has also been associated with slowing down gastric emptying, which, in turn, critically impacts the rate of absorption as evidenced by increased Tmax [50]. These changes can negatively impact the clinical efficacy of medications that require quick onset of action. Increased gastric pH can result from normal inter-occasion stomach pH variability (or in subjects with achlorhydria), which can impact the solubility of a weak base, such as ABZ (pKa 2.8). Model sensitivity analysis demonstrated that systemic exposure to ABZ’s active metabolite (ABZ_SO) was negatively influenced by the impact of increased gastric pH on the ABZ solubility [46,49,50]. In the absence of formulation influences, total drug solubility is a function of the intrinsic solubility and the solubility of the ionized species. For ABZ, a low soluble compound, total solubility depends on the degree of ionization, which depends on gastrointestinal pH, consistent with the Henderson–Hasselbalch equation [51]. Hence, the PBPK model predicted a decrease in ABZ_SO systemic exposure at increased gastric pH may be attributed to the deprotonation of ABZ to its free base, thereby, limiting the solubility and consequently the rate and extent of absorption of ABZ. This is of potential clinical concern since it is of particular importance for an anti-infective medication, such as ABZ, where reduced exposure may lead to the loss of therapeutic efficacy. This type of initial assessment, using PBPK absorption modeling, can potentially guide labeling requirements for a drug product and/or identify the required precautions for when patients will be prescribed the medication [52]. To mitigate the risk of high stomach pH on drug bioavailability, salt forms, and amorphous solid dispersion (ASD) formulations have been utilized [52,53]. These kinds of predictions informed the need for the formulation strategy for ABZ, where drug solubility would not be significantly impacted by changes in gastric pH.

Sensitivity analysis of mean gastric residence time showed that ABZ_SO exposure (Cmax and AUC) increased with increasing MRT. This can be attributed to the increased dissolution of ABZ in the acidic environment resulting from the prolonged residence of the drug in the stomach. At an MRT of 2.0 h, the model predicted a slight decrease in Cmax (with no change in AUC) reflecting the impact of delayed absorption (increased Tmax).

A gastro-retentive drug delivery system (gastric mucoadhesive tablet or multiunit particulate system (MUPS)) strategy is a potential option for weak basic compounds by allowing for extended residence on the stomach to promote solubilization of the drug before its transits to the intestine [46,54,55,56,57]. One of the reasons for developing this type of formulation system was to counteract a compound’s poor solubility at higher pH levels [54,56,58]. PBPK model sensitivity predictions showed a modest increase in exposure of ABZ_SO (~24%) with increased gastric MRT. This suggests that a drug, such as ABZ, which increases gastric retention through formulation science should not be pursued. Hence, in silico testing can be used upfront to screen potential formulation strategies and to identify those approaches that have the potential to enhance drug bioavailability.

The particle size (PS) of the API is a critical quality attribute, and changes in this parameter may affect drug performance in vivo [59]. This is especially important for BCS class II and IV compounds, including ABZ. Particle size reduction is one of the important initial techniques in the decision tree for improving the bioavailability of a poorly soluble drug. Stillhart et. al. observed improved simulated exposure of basmisanil with a reduction in particle size for lower doses, although no improved exposure was observed for higher doses, making it a critical attribute in the dug development decision tree [60]. The Advanced Dissolution, Absorption, and Metabolism (ADAM) model in Simcyp™ utilizes the following diffusion layer model (DLM), Equation (3), to characterize dissolution [61]:

(3) $$DR\left(t\right)=-NS\frac{D_{\left(eff\right)}}{h_{\left(eff\right)}}*4\pi r^{2}+h_{\left(eff\right)}*\left(S_{\left(surface\right)}-C_{\left(bulk\right)}\right)$$

where *N* is the number of particles, *S* is the scalar factor, *D_eff_* and *h_eff_* are the effective diffusion constant and effective diffusion layer thickness, respectively, r is the radius of the particle, and *S_(surface)_* and *C_b(bulk)_* are the solubilities at the particle surface and in the bulk, respectively. Dissolution from the drug particles depends on the surface area of the particle, and the diffusion layer thickness, which is modeled by the Hintz–Johnson relationship [62]. Absorption modeling predictions of the effect of API particle size have been previously explored and reported [63,64]. Decreasing the particle size increases the surface area and, thus, the dissolution of the drug. To understand the effect of different particle sizes on the bioavailability of ABZ_SO, sensitivity analysis was performed over a range from 0.25 µm to 200 µm, assuming the formulation comprised monodispersed particles. The results showed that reducing the particle size below 40 μm did not improve the systemic exposure of ABZ_SO. This suggested that drug release is initially dissolution rate limited but becomes solubility limited following the dissolution of the smaller particles. Thus, simulations with further decreases in particle size did not show improved pharmacokinetic parameters. Above 50 μm, predictions showed a decrease in exposure to ABZ_SO, consistent with a decrease in ABZ dissolution. From a formulation perspective, this sensitivity analysis indicates that reducing the particle size of ABZ below the micronized range is not a viable strategy to improve the bioavailability of ABZ_SO.

Sensitivity analysis confirmed that increasing ABZ solubility would improve ABZ_SO systemic exposure. PBPK modeling further predicted that exposure metrics for ABZ_SO would be significantly improved with increased supersaturation and decreased precipitation of ABZ in the intestine, the primary site of drug absorption. Hence, increasing the solubility of ABZ in the intestinal fluids accompanied by increased supersaturation and reduced precipitation creates a larger concentration gradient driving absorption. Kourentas et al. observed significant supersaturation of ABZ in the duodenum using an HPMC-based ABZ suspension formulation [24]. This suggests that the inclusion of supersaturation-promoting excipients is a potential formulation approach to enhance the bioavailability of ABZ_SO.

Alternatively, model simulations demonstrated that ABZ_SO systemic exposure could be improved by increasing the equilibrium solubility of ABZ. Accordingly, formulation strategies such as amorphous solid dispersions (ASDs) or lipid formulations may be appropriate to achieve this goal. ASDs incorporate surfactants/solubilizing agents and polymers that increase the drug solubility and prolong the supersaturation state [65,66]. ASDs have also been found to minimize the detrimental effect of high gastric pH on the solubility of a weak base compound [67]. The inclusion of digestible oils and surfactants in lipid-based formulations interferes with nucleation, and this approach can be explored as a way to develop efficient precipitation inhibitor formulations [16,68,69].

For a weak base such as ABZ, conventional solubility, and dissolution experiments will only provide a comparative understanding among prototype formulations. However, PBPK modeling simulations for ABZ demonstrate that in order to anticipate the in vivo performance of a formulation, it is extremely important to study the degree of supersaturation and precipitation rate because these factors determine both the amount of soluble drug and the time period that is it available for absorption in the intestine. Some of the major advantages of PBPK absorption modeling are that it captures the dynamic pH changes that occur throughout the GI tract and also considers the precipitation kinetics of the drug, which are critical in understanding the impact of formulation on the bioavailability of weak basic compounds. The approach used in this study builds upon previous investigations that combined in silico modeling with in vitro transfer experiments [9,28,30].

Overall, these analyses based on PBPK absorption modeling provided critical insight into which parameters to target when developing oral formulations for ABZ. Model predictions provide a foundation for future studies that can further validate the model by preparing prototype formulations of ABZ using various techniques (e.g., ASD and lipid-based systems) and characterizing their performance in terms of solubility and precipitation kinetics.

## 5. Conclusions

Understanding the critical quality attributes affecting the in vivo performance of a potential drug candidate prior to clinical testing would be beneficial in designing an oral formulation that can maximize efficacy by improving the rate and extent of absorption. In this research, absorption modeling combined with in vitro experiments provided an increased understanding of the complex in vivo performance of a low soluble prodrug, ABZ. PBPK absorption modeling with the Simcyp™ Simulator identified two critical parameters that drive the systemic exposure of its active metabolite, ABZ_SO: (1) gastric pH and (2) drug precipitation in the basic environment of the upper small intestine. Model sensitivity analysis inferred that reducing particle sizes would not improve ABZ_SO bioavailability and this strategy should be eliminated from the formulation decision tree. Alternatively, formulations containing excipients that either maintain the microenvironmental pH around ABZ to mitigate the impact of gastric pH changes on dissolution or can delay ABZ precipitation upon its transit into the intestinal environment should be considered.

## Figures and Tables

**Table 1:** Summary of the PBPK parameters for albendazole (substrate) and albendazole sulfoxide (primary metabolite) used in Simcyp™.

Albendazole (Substrate)			
Physiochemical Properties	Value		Ref/Comments
Molecular weight (g/mol)	265.33		[33]
Log Po: w	2.7		[34]
pKa (Ampholyte)			
pKa1	10.26		[35]
pKa2	2.8		[35]
B/P (User input)	0.55		Default
fu	0.156		Predicted
**Absorption**			
**Model (ADAM)**			
fuGut	1		Predicted
**Permeability parameters**			
Peff,man (10–4 cm/s)	8.03		Predicted
**Formulation parameters**			
**Solid Formulation (Diffusion layer Model)**			
Include bile salt-medicated component (Km:w)	Neutral: 2.924		Experimental (SIVA)
	Ion: 5.423		
Particle size distribution (polydispersed) (µm)	Weibull Distribution	Alpha: 1.014 Beta: 8.732	Experimental
	Mean Radius (µm)	7.52	
Intrinsic solubility (mg/mL)	0.012		Experimental (SIVA)
Critical Supersaturation Ratio	1		Experimental
Precipitation rate constant	1000 (max. value)		
**Distribution**			
Full PBPK model Vss (L/kg)	2.59		Predicted
**Elimination**			
Enzyme Kinetics CYP3A4			
Vmax (pmol/min/pmol)	369		
km (µM)	10.1		[36]
FMO3			
Vmax (pmol/min/pmol)	1103		
km (µM)	9.6		[36]
Gut lumen Clint (µL/h/g) of the total luminal content	2000		Optimized
**Albendazole sulfoxide (Primary metabolite)**			
Molecular weight (g/mol)	284.35		[37]
Log Po: w	1.17		[38]
pka (Ampholyte)			
pka1	9.79		[35]
pka2	0.2		
B/P (User input)	0.55		Default
fu	0.3		Literature
**Absorption**			
**Model (ADAM)**			
fuGut	0.36		Predicted
Peff,man (10–4 cm/s)	4.13		Predicted
**Distribution**			
Minimal PBPK Vss (L/kg)	2.3		Optimized
**Elimination**			
Oral clearance (L/h)	40		Optimized

B/P: blood to plasma ratio; fu: fraction unbound in plasma; fuGut: fraction unbound in enterocyte; Peff,man: effective permeability in humans; Clint: intrinsic clearance.

**Table 2:** Measured solubility profile of pure ABZ in various pH systems and biorelevant media at 37 ± 2.0 °C.

pH/Media	Solubility (mg/mL)
pH 1.2 HCl buffer	0.515 ± 0.045
pH 2.0 HCl buffer	0.025 ± 0.005
pH 4.5 Acetate buffer	0.008 ± 0.002
pH 6.8 Phosphate buffer	0.005 ± 0.001
FaSSGF (pH 1.6)	0.146 ± 0.012
FaSSIF (pH 6.5)	0.008 ± 0.001
FeSSIF (pH 5.0)	0.021 ± 0.004

**Table 3:** Observed and predicted pharmacokinetic parameters for ABZ and ABZ_SO.

	Albendazole	Albendazole Sulfoxide
C_max_ (Observed) (ng/mL)	9.55	193.5
C_max_ (Predicted) (ng/mL)	11.2	195.1
Fold error *	1.16	1.01
AUC_0–∞_ (observed) (ng/mL × h)	53.0	3475
AUC_0–∞_ (predicted) (ng/mL × h)	59.7	3405
Fold error *	1.13	0.97

* Fold error: predicted parameter/observed parameter.

## Data Availability

No new data were created.
